# Genetic mechanisms of knee osteoarthritis: a population-based longitudinal study

**DOI:** 10.1186/ar1835

**Published:** 2005-11-21

**Authors:** Changhai Ding, Flavia Cicuttini, Leigh Blizzard, Graeme Jones

**Affiliations:** 1 Menzies Research Institute, University of Tasmania, Hobart, Australia; 2 Department of Epidemiology and Preventive Medicine, Monash University Medical School, Melbourne, Australia

## Abstract

To describe the differences in knee structure and non-knee structural factors between offspring having at least one parent with a total knee replacement for severe primary knee osteoarthritis and age- and sex-matched controls with no family history of knee osteoarthritis, a population-based longitudinal study of 163 matched pairs (mean age 45 years, range 26 to 61) was performed at baseline and about 2 years later. Knee cartilage defect score (0 to 4), cartilage volume and bone size were determined with T1-weighted fat saturation magnetic resonance imaging. Body mass index (BMI), lower-limb muscle strength, knee pain, physical work capacity at 170 beats/minute (PWC170) and radiographic osteoarthritis were measured by standard protocols. In comparison with controls, offspring had higher annual knee cartilage loss (-3.1% versus -2.0% at medial tibial site, -1.9% versus -1.1% at lateral tibial site and -4.7% versus -3.7% at patellar site, all *P *< 0.05), a greater increase in medial cartilage defect score (+0.15 versus -0.01, *P *< 0.05) and a greater decline in PWC170 (-0.7 watts/kg versus -0.4 watts/kg, *P *< 0.01). There were no significant differences in change in BMI, lower-limb muscle strength, knee pain or tibial bone area between these two groups; however, the differences in knee cartilage loss and cartilage defect change decreased in magnitude and became non-significant after adjustment for baseline cartilage volume, tibial bone area, BMI and knee pain. This longitudinal study suggests that knee cartilage loss, change in cartilage defects and decrease in physical fitness all have roles in the development of knee osteoarthritis, which is most probably polygenic but may reflect a shared environment. Importantly, the cartilage changes are largely dependent on baseline differences in cartilage volume, tibial bone area, BMI and knee pain, suggesting that these factors might have a role in their initiation.

## Introduction

Knee osteoarthritis (OA) is a slowly developing chronic disease that has a multifactorial origin. Although several environmental factors including obesity, acute joint injury and occupational factors are important in its pathogenesis [1], a modest but significant genetic effect for knee radiographic osteoarthritis (ROA) has been reported in most studies [2-6] although the actual genes and mechanisms underlying this association are uncertain. A limitation of these studies is that radiographic measurement used in most of them provides only a broad-brush view of joint pathology because of its two-dimensional nature, semi-quantitative scoring system and/or inherent measurement error. Magnetic resonance imaging (MRI) can visualize knee joint structure directly and is recognised as a valid, accurate and reproducible tool for measuring knee cartilage volume [7-9], tibial bone surface area [7,10] and cartilage defects [11] and therefore has the potential to delineate early structural change in the knee. Recent data from our case control study suggested greater medial tibial bone area [12] and both more prevalent and more severe knee cartilage defects [13] but no difference in knee cartilage volume [12] in the offspring of individuals with severe knee OA in later life compared with controls, whereas tibial bone surface area [14], knee cartilage defects [13] and cartilage volume [14,15] have significant heritability in twin and sibling-pair studies. These results suggest that increases in tibial bone area and cartilage defects may be under genetic control. In contrast, cartilage volume is not an initiating factor even given cartilage loss is a key factor in established OA [16].

Non-knee structural components such as higher body mass index (BMI) and lower-limb muscle strength [12,14] might also have a role in the genetics of knee OA, although Manek and colleagues have suggested that obesity and OA are under separate genetic control [17]. It is unclear what the role of physical fitness is. The aim of this population-based longitudinal study was therefore to describe the two-year change in knee structure and non-knee structural factors between offspring having at least one parent with a total knee replacement for severe primary knee OA, and age- and sex-matched controls with no family history of knee OA.

## Materials and methods

### Subjects

This study was performed in southern Tasmania, primarily in the capital city of Hobart. The initial measurements were made during the period from June 2000 to December 2001, and the follow-up was conducted about two years later. Subjects were selected from two sources, as described previously [12]. Half (*n *= 163) of the 326 subjects were the adult children (offspring) of patients who had had a knee replacement performed for primary knee osteoarthritis at any Hobart hospital during the years 1996 to 2000. This diagnosis was confirmed by reference to the medical records of the orthopaedic surgeon and the original radiograph where possible. The family structure of each of the 163 offspring ranged through one offspring per family (*n *= 48), two offspring per family (35 families, *n *= 70), three offspring per family (9 families, *n *= 27), four offspring per family (3 families, *n *= 12) and six offspring per family (1 family, *n *= 6). The other half were controls selected at random from the most up-to-date version at the time (2000) of the roll of persons registered to vote in Southern Tasmania, a comprehensive population listing, and individually matched to cases by sex and to within 5 years of age. Those selected were eligible to participate if they had no parent with either a history of symptomatic knee OA or a knee replacement for OA.

To maximise data usefulness and minimise dropouts we rematched 15 of the original pairs according to age and sex. Subjects from either group were excluded on the basis of contraindication to MRI (including metal sutures, presence of shrapnel, iron filings in the eye and claustrophobia). No women were on hormone replacement therapy at the time of the study and medication use was uncommon. This study was approved by the Southern Tasmanian Health and Medical Human Research Ethics Committee, and all subjects provided informed written consent.

### Anthropometrics

Weight was measured to the nearest 0.1 kg (with shoes, socks and bulky clothing removed) with a single pair of electronic scales (Seca Delta Model 707), which were calibrated with a known weight at the beginning of each clinic. Height was measured to the nearest 0.1 cm (with shoes and socks removed) with a stadiometer. BMI (kg/m^2^) was calculated. Knee pain at baseline was assessed by questionnaire and was defined as pain for more than 24 hours in the past 12 months or daily pain on more than 30 days of the last year. Among the subjects with no knee pain at baseline, knee pain (on flat surface, going up or down stairs, at night, sitting or lying, and standing upright) was assessed by self-administered questionnaire with the WOMAC (Western Ontario and McMaster Universities Osteoarthritis) index, which ranges from 0 (no pain) to 9 (most severe pain) two years later. Incident knee pain was defined as having occurred if the total score (range 0 to 45) exceeded zero.

Objective measures of physical activity included measurement of muscle strength by dynamometry at the lower limb (involving both legs simultaneously). The subject was instructed in each technique before testing and each measure was performed twice. Repeatability estimates (Cronbach's α) were 0.91. The devices were calibrated by suspending known weights at regular intervals. Physical work capacity was also assessed with a bicycle ergometer [18]. Subjects were asked to cycle at a constant 60 r.p.m. for 3 minutes at three successively increasing but submaximal workloads. Heart rate was recorded, with an electric heart-rate monitor, at 1 minute intervals at each workload. Physical work capacity at 170 beats/minute (PWC170) was estimated by extrapolating the line of best fit to the three submaximal heart-rate-work-capacity data points to estimate PWC170. The PWC170 was not considered a technically adequate measure unless subjects had spent a minimum of 2 minutes at each workload and the pulse rate increased by at least 5 beats/minute with increasing workloads. Repeatability was not assessed in our subjects but has previously been reported to be as high as 0.92 [18].

### X-ray

A standing anteroposterior semiflexed view of the right knee was performed in all subjects at baseline and scored individually for osteophytes and joint space narrowing as described previously [19].

### Knee cartilage volume measurement

MRI scans of the right knees were performed in the cross-sectional and follow-up study. Knees were imaged in the sagittal plane on a 1.5 T whole-body magnetic resonance unit (Picker) with use of a commercial transmit-receive extremity coil. The following image sequence was used: a T1-weighted fat saturation three-dimensional gradient recall acquisition in the steady state; flip angle 55°; repetition time 58 ms; echo time 12 ms; field of view 16 cm; 60 partitions; 512 × 512 matrix; acquisition time 11 minutes 56 s; one acquisition. Sagittal images were obtained at a partition thickness of 1.5 mm and an in-plane resolution of 0.31 × 0.31 (512 × 512 pixels). Knee cartilage volume was determined by image processing on an independent workstation using Osiris (University of Geneva) as described previously [7]. The volumes of individual cartilage plates (medial tibial, lateral tibial and patella) were isolated from the total volume by manually drawing disarticulation contours around the cartilage boundaries on a section by section basis. These data were then resampled by means of bilinear and cubic interpolation (area of 312 μm × 312 μm, 1.5 mm thickness; continuous sections) for the final three-dimensional rendering. The volume of the particular cartilage plate was then determined by summing all the pertinent voxels within the resultant binary volume.

Femoral cartilage volume was not assessed because we have published that two tibial sites and the patella site are correlated strongly with this site [20]. Using this method we had high intraobserver and interobserver reproducibility. The coefficient of variation for cartilage volume measures was 2.1% for medial tibial, 2.2% for lateral tibial and 2.6% for patella [7].

### Knee bone size measurement

Knee tibial plateau bone area and patellar bone volume were determined by means of image processing on an independent workstation using Osiris as described previously [7,10]. To transform the images to the axial plane, the Analyse Software package developed by the Mayo Clinic was employed. The coefficients of variation for these measures in our hands are 2.2 to 2.6% [7].

### Assessment of cartilage defects

Cartilage defects were graded as described previously [11] on two occasions of MRI sagittal scans at medial tibial, lateral tibial and patellar sites as follows: grade 0, normal cartilage; grade 1, focal blistering and intracartilaginous low-signal intensity area with an intact surface and bottom; grade 2, irregularities on the surface or bottom and loss of thickness of less than 50%; grade 3, deep ulceration with loss of thickness of more than 50%; grade 4, full-thickness chondral wear with exposure of subchondral bone. The cartilage was considered to be normal if the band of intermediate signal intensity had a uniform thickness. The cartilage defects were regraded 1 month later and the average scores of cartilage defects at medial tibiofemoral (0 to 8), lateral tibiofemoral (0 to 8) and patellar (0 to 4) sites were used in the study. Intraobserver reliability (expressed as intraclass correlation coefficient) was 0.89 to 0.94 and interobserver reliability was 0.85 to 0.93 [11].

### Data analysis

Rates of change in cartilage volume were calculated both as the absolute change per annum, (*v*_1 _- *v*_0_)/*t*, and as the percentage change per annum, 100(*v*_1 _- *v*_0_)/*v*_0_*t*, where *v*_0 _is cartilage volume at baseline, *v*_1 _is cartilage volume at follow-up and *t *is the time between scans in years.

A combination of paired *t* tests, McNemar tests and linear regression were used for the analyses of this matched dataset. To take the relatedness of offspring subjects into account, family clusters of offspring–control pairs with related offspring were identified, and robust variance estimates based on the clustering were obtained. This method relaxed the untenable assumption that all offspring–control pairs were independent and required only that the family clusters were independent. The robust standard errors, which in nearly every case were substantially larger than estimates ignoring the clustering, were used to calculate the 95% confidence intervals (CIs) reported. The selection of covariates used in multivariable analyses was determined by a need to understand possible mechanisms of effect. All factors that were different at baseline (BMI, muscle strength, knee pain, chondral defects and tibial bone area) were automatically included [12,13]. Other factors that were considered biologically important, such as cartilage volume, were also included.

For the regression analyses, differences between offspring and control in annual changes in knee structure and non-knee structural factors were calculated and regressed on differences at baseline in knee cartilage volume or cartilage defect scores, BMI, prevalence of knee pain, knee bone size, lower-limb muscle strength and ROA. We routinely checked to determine whether model fit was significantly improved by adding product terms between pairs of predictors, and included those that were. There was no collinearity between chondral defects and cartilage volume. The differences in annual changes in cartilage volume and changes in cartilage defect scores were further adjusted for each other. Standard diagnostic checks of model fit and residuals were made routinely, and data points with large residuals and/or high influence were investigated for data errors (but we did not exclude any subjects, because all corrected values were considered plausible). A 10% change in the coefficient after adjustment for a variable was accepted as providing evidence of a factor acting as an intermediate variable. *P *< 0.05 (two-tailed) or a 95% CI not including the null point was regarded as statistically significant. Adjustment for multiple comparisons was not performed. All statistical analyses were performed with STATA version 8.0 (Statistical Software, Release 8, 2003; Stata Corporation, College Station, TX, USA).

## Results

A total of 326 subjects (136 males, 190 females) comprising 163 offspring and 163 age- and sex-matched controls completed the study (87% of those originally studied). There were no significant differences at baseline in any factor between completers and non-completers (data not shown). This was a young sample with an average age of 45 years (range 26 to 61) at baseline. The average time between visits was 2.3 years (range 1.8 to 2.6). Characteristics of the subjects are presented in Table 1. Offspring were heavier than controls at baseline, with less lower-limb muscle strength and a greater prevalence of knee pain, but the changes in these factors during follow-up were similar. There was no difference in PWC170 at baseline between the two groups, but offspring had significantly greater decrease in PWC170 during follow-up.

Table 2 documents the differences in rate of change in cartilage volume and tibial bone area, and changes in cartilage defect scores, between offspring and controls. In offspring, absolute loss in medial tibial and total cartilage volume per annum, percentage loss in medial tibial, lateral tibial, patellar and total cartilage volume per annum and change in medial cartilage defect score were significantly higher than in controls (all *P *< 0.05). In contrast, no significant differences between offspring and controls were observed in changes in tibial bone area.

Table 3 details the multivariable analyses for differences between offspring and controls. After adjustment for differences in baseline cartilage volume, the differences in percentage changes in medial and lateral tibial cartilage volume became non-significant and decreased by 27% and 30%, respectively, whereas the difference in percentage change in patellar cartilage volume remained unchanged. The differences in percentage changes in cartilage volume decreased in magnitude and became non-significant after adjustment for differences in BMI (19 to 24%), knee pain (13 to 30%), corresponding bone size (27 to 40% for changes in tibial and total cartilage volume) and changes in corresponding cartilage defect scores (23 to 33% for changes in medial tibial, patellar and total cartilage volumes), respectively. In total, the coefficients for percentage change in cartilage volume decreased by 44 to 75% after adjustment for all of the above factors.

The differences in medial tibial, patellar and total cartilage defect changes increased and/or became significant after adjustment for differences in baseline cartilage defect scores (Table 3). However, these differences decreased in magnitude after adjustment for differences in baseline BMI (14 to 22%), knee pain (12 to 19% for change in medial and total cartilage defects), corresponding bone size (11 to 38%) and changes in corresponding cartilage volume (44 to 58%).

The difference in change in physical work capacity (β = -0.29, 95% CI -0.50, -0.09) decreased in magnitude after adjustment for baseline physical work capacity (β = -0.20, 95% CI -0.35, -0.04), BMI (β = -0.21, 95% CI -0.42, +0.01) and knee pain (β = -0.26, 95% CI -0.48, -0.03). The difference was increased slightly by adjustment for change in BMI (β = -0.31, 95% CI -0.51, -0.11). After adjustment for the difference in change in physical work capacity, the coefficients for change in knee cartilage volume decreased by between 28% and 58%.

Results were largely unchanged after adjustment for lower-limb muscle strength, past knee injury and ROA (data not shown).

## Discussion

This is the first longitudinal study to examine differences in knee structural components and physical measures between offspring of those with severe knee OA in later life and population controls without this history. Offspring had higher rates of knee cartilage loss in all compartments, more cartilage defect development in medial tibiofemoral and patellar compartments and a greater decline in physical work capacity than controls during two years. Interestingly, these differences seemed largely to be mediated with differences in baseline cartilage measures, tibial bone area, BMI and knee pain.

Cartilage loss is the hallmark of established OA, and 60% of cartilage is lost by end-stage knee OA [21,22]. Cartilage loss is of the order of 5% per annum in established knee OA [16] and is a predictor of knee replacement [22]. However, relatively little is known about the determinants of cartilage loss. Our cross-sectional study found a non-significant trend to higher tibial cartilage volume in the offspring of those with severe knee OA compared with controls [12]. Our method cannot distinguish normal from swollen cartilage at a given point in time but this suggests that cartilage volume may increase in the early stages of OA due to cartilage swelling [23] and led us to speculate there would be a higher rate of cartilage loss in the offspring over time [12]. This was confirmed by the present study, in which there was a consistently higher rate of cartilage loss in all knee compartments. This is consistent with our recent observation in sibling pairs that the rate of cartilage loss had high heritability, especially at the medial tibial site [24]. The magnitude of these differences in cartilage loss was substantial and of clinical significance: for example, if rates of loss are constant it can be estimated that controls at mid-life will lose the amount of medial tibial cartilage required to reach end-stage OA in 30 years, but this deterioration will take only 19 years for offspring. These differences in medial and lateral tibial cartilage loss decreased and became non-significant after adjustment for differences in baseline cartilage volume, suggesting that early swelling of cartilage will be followed by higher rates of tibial cartilage loss over time.

Knee cartilage defects are very common [11] but little is known about their relevance, causes or natural history. We have reported in a cross-sectional study that age [25], BMI [26], ROA, decreased cartilage volume and increased urinary levels of type II collagen breakdown [11] are associated with the prevalence and severity of knee cartilage defects, suggesting an important role for knee cartilage defects in early knee OA. In the initial cross-sectional comparison we reported a higher prevalence and severity of cartilage defects in offspring [13]. Consistent with this, the present study found more knee cartilage defect development in offspring in medial, patellar and total compartments. The change in knee cartilage defects was shared in part with the change in knee cartilage volume, which is not unexpected. Again, these changes were dependent on initial differences, especially increased tibial surface area, suggesting that this might initiate cartilage defects.

Subchondral bone is important in the initiation and progression of knee OA [27,28]. We have reported that subchondral bone size increased with age [25] and BMI [26], and was markedly increased in subjects with osteophytes [19] and correlated with the prevalence and severity of knee cartilage defects [11]. Tibial bone area is also heritable mainly through body size [14], and baseline medial tibial bone area was modestly but significantly higher in the offspring of those with severe knee OA in comparison with controls [12]. In this longitudinal study we found no significant change in both medial and lateral tibial bone area in offspring. This might actually be true or might reflect measurement error in the assessment of change in knee bone size over a relatively short study period. However, baseline tibial bone area differences were relevant for both cartilage volume and defect changes over time, suggesting that early subchondral bone expansion can partly explain the genetic contribution to cartilage loss and the development of cartilage defects.

As reported previously [12], offspring weighed more, had a higher BMI, weaker lower-limb muscle strength and a higher prevalence of knee pain at baseline. This suggests to us that these factors might also contribute to genetic mechanisms of knee OA but might also have been due to selection bias (see below). During two years of follow-up we failed to confirm these changes, despite a greater decrease in cartilage volume and greater increase in cartilage defects in offspring compared with controls. However, adjusting for baseline differences in BMI and prevalent knee pain suggested that these factors did explain some of the changes in cartilage. Furthermore, this study cannot exclude the possibility of an explanation involving physical activity and fitness, because we found that offspring had a higher rate of decrease in PWC170 than controls, and adjusting for offspring–control differences in change in PWC170 greatly reduced the estimated offspring–control differences in loss of cartilage volume. Previous studies have shown that PWC170, a measure of cardiorespiratory fitness, is associated with obesity [29] and bone mineral density at the femoral neck and spine [30] in girls, but the role of PWC170 in the pathogenesis of knee OA is novel. The offspring–control difference in this change was dependent on differences in baseline BMI and knee pain, suggesting the genetic contribution to BMI and knee pain is also relevant to the genetic contribution to change in physical fitness.

This study has several potential limitations. First, the two years of follow-up in this study may have been too short to enable us to detect the hypothesised differences in change in BMI, lower-limb muscle strength and knee pain. A study with a longer period of follow-up might be required for this. Second, the low rate of participation by controls in this study (40% at baseline) raises the possibility that selection bias might have obscured the hypothesised changes. Although loss to follow-up is not of major concern because participation at follow-up was high (87%), we have previously [12] cautioned that the offspring–control differences observed at baseline might be due at least in part to the controls being relatively healthy individuals who participated because they were unusually interested in their health. Third, we did not have radiographic measurement for the follow-up study. While X-ray is the gold standard for the definition of OA (in combination with pain), changes occur slowly, possibly because they are being greatly diluted by measurement error. This, combined with the young age and low prevalence of ROA in this sample, led us to elect not to perform follow-up radiographs in this study. Furthermore, adjustment for baseline ROA did not alter the results, suggesting that it might not be an ideal measure in early disease anyway. Fourth, measurement error may influence results. However, knee cartilage volume, scoring of cartilage defects and measurements of bone size are all highly reproducible, suggesting that this is unlikely. Finally, some of the offspring were related. We took this into account in analysis, but their relatedness reduced our power to detect changes over time.

## Conclusion

This longitudinal study suggests that knee cartilage loss, change in cartilage defects and decrease in physical fitness all have roles in the development of knee OA, which is most probably polygenic but might reflect shared environment. Importantly, the cartilage changes are largely dependent on baseline differences in cartilage volume, tibial bone area, BMI and knee pain, suggesting that these factors might have a role in their initiation.

## Abbreviations

BMI = body mass index; CI = confidence interval; MRI = magnetic resonance imaging; OA = osteoarthritis; PWC170 = physical work capacity at 170 beats/minute; ROA = radiographic osteoarthritis.

## Competing interests

The authors declare that they have no competing interests.

## Authors' contributions

GJ, FC and CD participated in the design of the study. CD performed the measurement of cartilage volume, cartilage defects and bone size and drafted the manuscript. CD and LB performed the statistical analysis. GJ, FC and LB reviewed the manuscript. All authors read and approved the final manuscript.

## Figures and Tables

**Table 1:** Characteristics of participants (*n *= 326)

Characteristic	Offspring (*n *= 163)	Controls (*n *= 163)	*P*
Values at baseline (2000–2001)			
Age (years)	45.1 ± 6.5	45.2 ± 6.5	0.87
Height (cm)	169 ± 8	169 ± 9	0.84
Weight (kg)	80 ± 16	75 ± 14	0.04
BMI (kg/m^2^)	27.9 ± 5.1	26.2 ± 4.3	0.02
Prevalence of knee pain (%)	47	22	<0.01^a^
Lower-limb muscle strength (kg)	127 ± 49	135 ± 50	0.006
PWC170 (W/kg)	2.94 ± 1.13	2.91 ± 0.99	0.77
Change during two-year follow-up			
Change in weight (kg)	1.2 ± 4.4	1.3 ± 5.0	0.89
Change in BMI (kg/m^2^)	0.6 ± 1.5	0.7 ± 1.8	0.58
Incidence of knee pain (%)	47	41	0.35^a^
Change in lower-limb muscle strength (kg)	-8.5 ± 24.6	-10.7 ± 28.0	0.47
Change in PWC170 (W/kg)	-0.7 ± 1.0	-0.4 ± 0.9	<0.01

Where errors are shown, results are means ± SD. BMI, body mass index; PWC170, physical work capacity at 170 beats/minute. ^a^McNemar tests; others are paired *t* tests.

**Table 2:** Comparison of annual change knee structure between offspring and controls

Parameter	Annual change		*P*
	Offspring	Controls	
Medial tibial cartilage volume (mm^3^)	-74 ± 88	-51 ± 103	0.027
Lateral tibial cartilage volume (mm^3^)	-52 ± 88	-35 ± 98	0.115
Patellar cartilage volume (mm^3^)	-167 ± 137	-138 ± 153	0.059
Total cartilage volume (mm^3^)	-292 ± 246	-225 ± 284	0.023
Medial tibial cartilage volume (%)	-3.1 (3.6)	-2.0 (4.5)	0.019
Lateral tibial cartilage volume (%)	-1.9 (3.1)	-1.1 (3.7)	0.045
Patellar cartilage volume (%)	-4.7 (3.7)	-3.7 (3.8)	0.019
Total cartilage volume (%)	-3.4 (2.7)	-2.5 (3.1)	0.009
Medial cartilage defect score	0.15 ± 0.70	-0.01 ± 0.64	0.031
Lateral cartilage defect score	0.02 ± 0.65	0.02 ± 0.76	0.937
Patellar cartilage defect score	0.25 ± 0.67	0.16 ± 0.64	0.213
Total cartilage defect score	0.42 ± 1.42	0.16 ± 1.50	0.100
Medial tibial bone area (mm^2^)	10 ± 38	8 ± 30	0.607
Lateral tibial bone area (mm^2^)	0.0 ± 27	-0.5 ± 43	0.905
Medial tibial bone area (%)	0.6 (2.0)	0.4 (1.7)	0.411
Lateral tibial bone area (%)	0.1 (3.6)	-0.0 (2.3)	0.682

Where errors are shown, results are means ± SD. Numbers in parentheses are also SD.

**Table 3:** Multivariable analyses of differences between offspring and controls in changes in cartilage measures

Outcome factor	Univariable analysis	β and 95% confidence interval					
		Cartilage^a^	Cartilage^a ^and BMI	Knee pain	Bone size^b^	Other outcome^c^	All factors
Differences in annual change in cartilage volume							
Medial tibial (%)	**-1.06 (-2.01,-0.10)**	-0.77 (-1.61,+0.08)	-0.62 (-1.46,+0.23)	-0.89 (-1.88,+0.10)	-0.64 (-1.60,+0.31)	-0.71 (-1.57,+0.15)	-0.27 (-1.14,+0.60)
Lateral tibial (%)	**-0.79 (-1.60,-0.00)**	-0.55 (-1.32,+0.21)	-0.44 (-1.22,+0.33)	-0.55 (-1.43,+0.33)	-0.52 (-1.33,+0.29)	-0.75 (-1.59,+0.07)	-0.32 (-1.21,+0.56)
Patellar (%)	**-0.98 (-1.83,-0.12)**	**-0.96 (-1.78,-0.15)**	-0.74 (-1.56,+0.09)	-0.85 (-1.76,+0.06)	-1.01 (-1.87,-0.17)	-0.75 (-1.56,+0.06)	-0.55 (-1.37,+0.26)
Total (%)	**-0.86 (-1.54,-0.17)**	**-0.71 (-1.33,-0.10)**	-0.54 (-1.17,+0.09)	-0.69 (-1.40,+0.02)	-0.63 (-1.34,+0.07)	-0.60 (-1.23,+0.04)	-0.28 (-0.94,+0.38)
Differences in annual change in cartilage defects							
Medial (number)	**0.16 (0.00,0.32)**	**0.22 (0.07,0.38)**	**0.19 (0.05,0.34)**	0.13 (-0.04,+0.31)	0.11 (-0.06,+0.27)	0.09 (-0.05,+0.23)	0.11 (-0.04,+0.26)
Patella (number)	0.09 (-0.05,+0.24)	**0.14 (0.00,0.29)**	0.12 (-0.03,+0.26)	0.09 (-0.06,+0.25)	0.08 (-0.06,+0.23)	0.05 (-0.09,+0.20)	0.07 (-0.07,+0.22)
Total (number)	0.26 (-0.06,+0.59)	**0.41 (0.06,0.76)**	0.32 (-0.01,+0.66)	0.23 (-0.12,+0.59)	0.16 (-0.17,+0.48)	0.11 (-0.19,+0.40)	0.19 (-0.13,+0.50)

Bold denotes statistical significance. ^a^Adjusted for differences at baseline in cartilage volume (analyses of differences in change in cartilage volume) or in cartilage defects (analyses of differences in change in cartilage defects); ^b^adjusted for differences at baseline in bone size at this site; ^c^adjusted for differences at baseline in annual change in cartilage defects (analyses of differences in change in cartilage volume) or in annual change in cartilage volume (analyses of differences in change in cartilage defects).
